# From Data to Design: Constructing Scapula and Hip Bone Through Online Datasets, Open-Source Software and 3D Printers

**DOI:** 10.7759/cureus.60212

**Published:** 2024-05-13

**Authors:** Dharam S Rathia, Vijay K Konuri

**Affiliations:** 1 Department of Anatomy, All India Institute of Medical Sciences, Raipur, Raipur, IND

**Keywords:** image segmentation, 3d slicer, innominate, scapula, ‎3d printing

## Abstract

Introduction

Human skeletons and bones are essential for medical and allied sciences students. Nowadays, it is becoming challenging to procure bone legally, resulting in medical students' inadequacy of bone. Plaster-of-Paris or resin bone models sold on the market are less detailed than real bones.

Aims and objectives

This study aims to create a three-dimensional (3D)-printed human bone model with free, open-source software and fused deposition modelling (FDM) 3D printers, compare its accuracy with the original bones and validate it with a textbook description.

Methods

Scapula and hip bone models were produced using open-source software 3D Slicer from computed tomography (CT) data from the “New Mexico Decedent Image Database”. After automated segmentation, bones were edited manually with tools in a 3D Slicer (https://www.slicer.org/) and Meshmixer software (Autodesk Inc., San Rafael, California, United States) and 3D bone models were printed using polylactic (PLA) filament.

Results and discussions

3D digital models of both bones were successfully created with the maximum possible accuracy with an FDM 3D printer. A 3D digital replica of the scapula obtained after segmentation retained most anatomical features except for the glenoid cavity, as the head of the humerus obscured the glenoid cavity. The 3D digital skeleton of the hip bone retained all anatomical features except articulating surfaces, such as the acetabulum and auricular surface ilium, which were obscured by the head of the femur and sacrum. A few morphological features of both bones differed from the original dry bone, and a few finer details were unclear in the iliac fossa and ischium. After manual editing and post-processing, the final physical model obtained has all the features.

Conclusions

We conclude that it is possible to produce anatomically accurate models with the greatest possible resemblance and accuracy to the original bones using free and open-source data with an FDM 3D printer.

## Introduction

Human skeletons and bones are essential for medical and allied sciences students [1-3]. In the early 2000s, the buying and selling of human skeletal material were prohibited in many countries [4]. Since 1985, the Indian government has imposed export prohibitions following a thorough analysis of the illegal bone trade [5]. The legal market fails to meet the demand, resulting in the use of illicit sources [6]. Even medical colleges in India have bone sets, and they are inadequate for a large number of students [2]. To address the ethical concerns and inadequacy of human bone, alternatives such as three-dimensional (3D) images, plastic models and wooden skull models have been explored as substitutes for human bones in teaching anatomy [6-8]. Plaster-of-Paris, or resin bone models that are sold in the market, are not as detailed as those of real bones [9]. Innovations like the Skeleton Virtual reality application have also been introduced to enhance students' learning and engagement in studying anatomy, but lack haptic feelings [10]. One helpful tool for modernising anatomy education is the creation of 3D-printed models that are anatomically accurate [11]. 3D printing or additive manufacturing (AM) is the layer-by-layer creation of a physical object from a 3D computer model [12]. Detailed methodologies have been developed to create optimized models of bone for 3D printing [13,14]. Here, we provide a similar method of making a 3D-printed anatomical model of the human scapula and innominate or hip bone using the open-source software 3D Slicer (https://www.slicer.org/), Meshmixer (Autodesk Inc., San Rafael, California, United States), and fused deposition modelling (FDM) 3D printers to observe the accuracy of gross anatomical features. This study aims to create a 3D-printed human bone model with free, open-source software and FDM 3D printers, compare its accuracy with the original bones and validate it with a textbook.

## Materials and methods

Radiological data source

Computed tomography (CT), Digital Imaging and Communications in Medicine (DICOM) data file, the international standard for medical images and related information of the individual were downloaded from the "New Mexico Decedent Image Database" [15].

Creating 3D model using 3D Slicer

**Figure 1:**
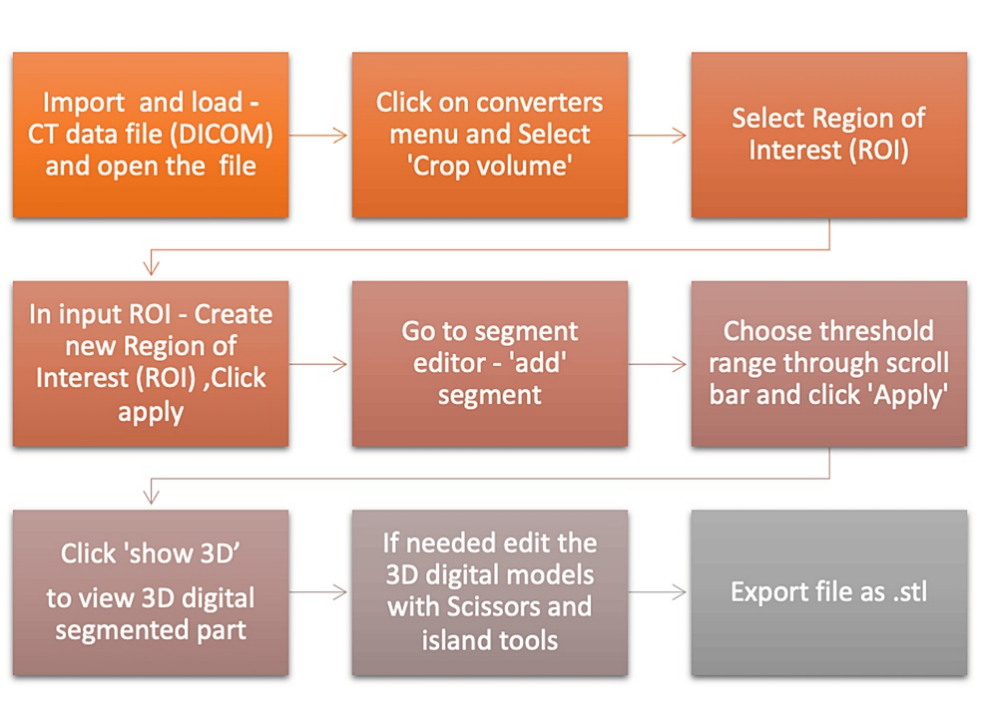
Process of image segmentation using 3D Slicer software from CT data Image created by Rathia DS and Konuri VK.

The segmentation of CT scan data of a patient from the database, image series for the upper limb region and torso, was done using open-source 3D Slicer software (Version 6.6.2) [16]. At first, we segmented the scapula using the thin bone "H.D.-UXT CT" image series. Before segmentation, the new region of interest (ROI) was selected with a converter menu and crop volume. Then, segmentation was done by the segment editor by selecting the threshold range 128-2976 (Figure 1; Table 1).

**Table 1:** CT image source and segmentation parameter

S. No.	CT image series description	Bone	Sex	3D Slicer threshold range, Max-Min
1	Thin bone HD-UXT	Scapula	Male	128-2976
2	Thin ST torso	Hip bone	Male	232-2976

Segmented regions were visualised by clicking the "Show 3D" icon. The individual left side scapula was isolated with editing tools like an island and a scissor in the segment editor. After segmentation, part of the humerus remains attached to the bone, and the surface of the bone shows a few unwanted spikes and holes. These parts were removed using an editing tool in 3D Slicer and Meshmixer software. Finally, the stereolithography or standard triangulation language (.stl) file of the bone model was exported and saved. The innominate or hip bone was segmented using the "Thin S.T. torso" CT image series. Like scapula segmentation, the new ROI was created with a converter menu and crop volume. Then, with the segment editor, manually segmented the left hip bone by selecting threshold range 232-2976 (Table 1, Figure 1). The "Show 3D" icon was used to visualise a segmented region in a 3D view. An individual hip bone was isolated from the ROI using an island and scissor tool. The .stl file of bone was exported and saved. Then, the file is inspected and corrected using Meshmixer software (Version 3.5.474). Further editing of both bone models was done with Meshmixer software to smoothen the articulating surface and remove the head of the humerus, femur and sacrum attached.

Creating physical 3D models using FDM 3D printers

The .stl file was converted into a geometric code (G codes) file using a 3D Slicer Crura software (version 16.04.6.). Models were optimised and reduced in size for printer-specific requirements (Table 2).

**Table 2:** Parameter and materials used for 3D printing bones PLA: polylactic filament

Model	Original dimension of bone in milimeters (mm)			Dimension optimised for printing uniform scaling in milimeters (mm)			Print time in hours	Material used (in grams)	PLA filament length in meters (m)
	x	y	z	x	y	z			
Scapula	119.603	96.733	172.463	120	80.14	57.905	6.15	53	17.69
Hip bone	126.365	136.293	246.635	130	78	60.8	7.9	49	16

**Figure 2:**
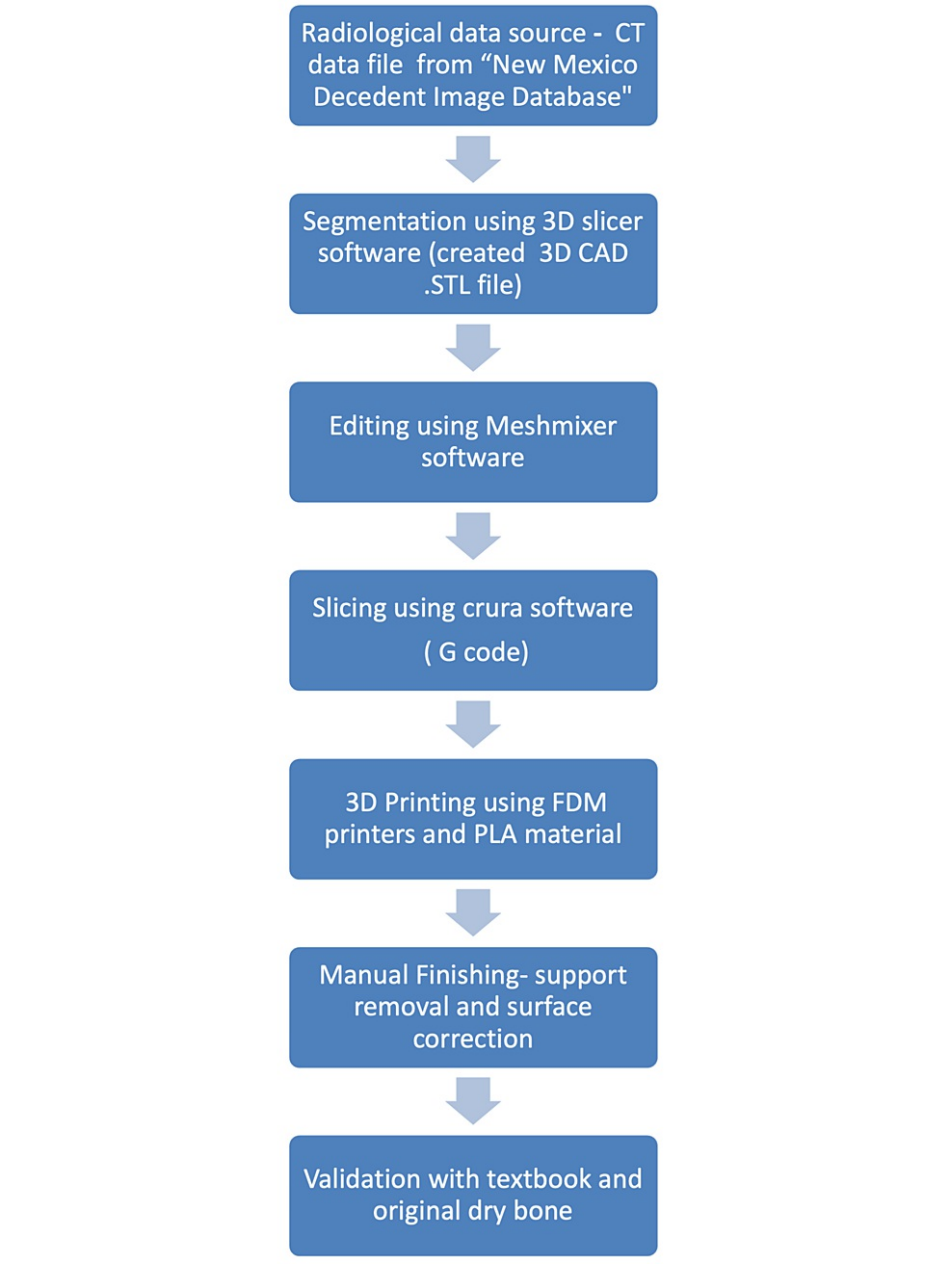
Flow chart showing the step-by-step process of creating 3D models Image created by Rathia DS and Konuri VK. FDM: fused deposition modelling; PLA: polylactic filament

Then, with the help of a 3D computer model, it was converted into a physical object using the FDM or fused filament fabrication (FFF) 3D printer (Pratham 3.0; Make3d.in, Surat, India). Using commercially available common 3D printing materials, polylactic (PLA) 3D models were created at 80% infill density. Post-processing, like surface smoothening and support removal, was done using a hand sander and drill. The models were compared with original dry bones and text descriptions from the anatomy book [17]. The following flowchart shows the whole process of our study flow chart of study (Figure 2).

## Results

**Figure 3:**
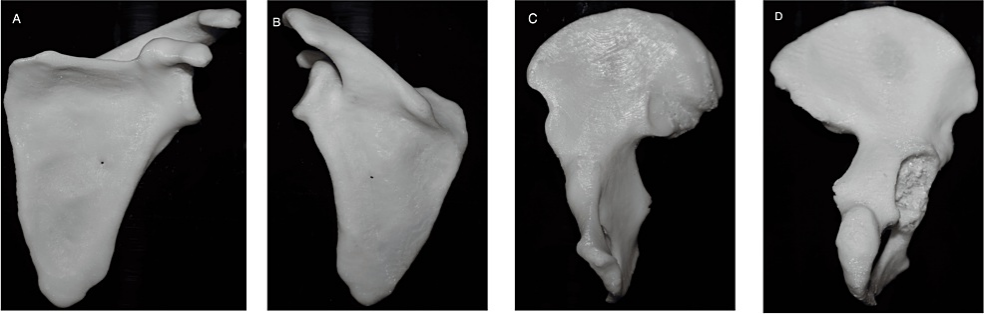
3D printed bone of scapula: (A) anterior view, (B) posterior view; Hip bone: (C) medial view, (D) lateral view Image created by Rathia DS and Konuri VK.

With the help of 3D Slicer software, we segmented the scapula and hip bone. The model of the scapula obtained lacks anatomical accuracy over the surface. A few morphological features can not be segmented like the original dry bone, and a few finer details over the surface of the supraspinous and interspinous fossa were uneven. The anatomical features of the final 3D scapula digital and physical models printed with a 3D printer were similar to the original dry bone and feature mentioned in the textbook (Figure 3; Table 3) [17].

**Table 3:** Anatomical features of scapula and comments on the accuracy of the model

S. No.	List of anatomical features of scapula	Anatomical accuracy of digital model (.stl)	Anatomical accuracy of 3D printed physical model after post-processing
1	Ventral surface	Accurate few spikes like projection	Accurate
2	Dorsal surface	Accurate small, hole	Accurate
3	Spine	Accurate	Accurate
4	Coracoid process	Accurate	Accurate
5	Acromion	Accurate	Accurate
6	Glenoid fossa	Surface with attached humerus	Accurate
7	Axillary margin	Accurate	Accurate
8	Medial angle	Accurate few spikes	Accurate
9	Vertebral margin	Accurate	Accurate

The model of hip bone obtained too had few faint anatomical features over the gluteal surface and ischium when compared with dry bones. A few morphological features differed from the original dry bone, and a few finer details were unclear. After segmentation, the articulating surface was not visible in the acetabular cavity. Part of the head of the femur and part of the sacrum remain attached to the bone, and the bone shows a few unwanted spikes and holes. These parts were removed using an editing tool in 3D Slicer and Meshmixer software. Finally, the anatomical features of the hip bone 3D digital model obtained after editing and physical models printed with a 3D printer were similar to the original dry bone and feature mentioned in the textbook (Table 4) [17].

**Table 4:** Anatomical features of the hip bone and comments on the accuracy

S. No.	Anatomical features of hip bone or innominate		Anatomical accuracy of digital model (.stl)	Anatomical accuracy of 3D printed physical model after post-processing
1	Ilium	Iliac crest	Accurate	Accurate
		Greater sciatic notch	Accurate	Accurate
		Lesser sciatic notch	Accurate	Accurate
		Iliac tuberosity	Accurate	Accurate
		Preauricular sulcus	Accurate	Accurate
		Iliac fossa	Accurate	Accurate
		Auricular surface	Obscured with attachment of sacrum	Accurate
		Anterior superior and inferior spines	Accurate	Accurate
2	Ischium	Ischial tuberosity	The surface feature not appreciable	Accurate
3	Pubis	Pubic symphyses	Accurate	Accurate
4	Structures formed by the intersection of the three bones of the innominate:	Obturator foramen	Accurate	Accurate
		Acetabulum Lunate fossa	Obscured by the head of the humerus need digital and manual correction after printing	Fragment of bone remains in the fossa not clearly appreciable

## Discussion

Research summary

Bone models of the scapula and hip bone were produced using open-source software 3D Slicer from CT data obtained from the "New Mexico Decedent Image Database" [15]. 3D Slicer is a free, open-source software for visualising, processing, segmenting, registering, and analysing medical, biomedical, and other 3D images and meshes and planning and navigating image-guided procedures [16]. Although the software used in the study is not meant for clinical use, it helps segment anatomical regions for research and education [16]. Segmentation is the division of magnetic resonance (MR) or CT images of an organ into distinct anatomical structures or segments of different tissue types done manually and automated [18]. After automated segmentation, bones were edited manually with tools in a 3D Slicer and Meshmixer and 3D prints were printed using PLA filament. PLA is cost-effective, is generally easier to work with, and produces more aesthetic-looking parts [19].

Interpretation of findings

We aimed to test the capability of free and open-source software to create bone models. After a few minor edits, the outcome showed that the artificial bone scapula and innominate created were anatomically as accurate as real dry bones described in the textbook. The surface and features were edited using tools in 3D Slicer and Meshmixer. The final physical model obtained after manual post-processing had most of the features and was accurate. The 3D digital skeleton of the hip bone retained all anatomical features except articulating surfaces, such as the acetabulum and auricular surface ilium, which were obscured by the head of the femur and sacrum. We found this method a potential alternative solution for creating accurate human bones. A study used and contrasted three distinct methods - 3D scanning, photogrammetry, and micro-CT - to create a digital 3D zygomatic bone and micro-CT was found to be the most effective method for achieving morphological accuracy [11]. A similar study utilizing the same free software, the 3D Slicer and the New Mexico Decedent Image Database successfully generated precise models of 2D human bones of the foot [13]. 3D-printed anatomical models were created to study frogs for anatomy education after 3D scanning and printing skeletal tissues [20]. As per the ethical concern and inadequacy that appeared in the various research articles, accurate bone in an adequate number can be produced using 3D printing bone models; it is an ethically potential solution for medical students [1,2,4,6]. Many studies have already mentioned the utility of 3D printed models for anatomy education for improving student engagement and skeletal tissue for medical and biology education [3,20,21].

Limitations and implications

Although we successfully created anatomically accurate artificial bones, it is limited by a few unreproduced surface features. These can be resolved by editing with individual experts in computer-assisted design (CAD) designing, and more accurate models can be produced as we desire pathological models. With 3D segmentation and printing, apart from creating more units of bone models for students' demonstration, we can also produce rare anatomical models with variations and pathologies, accidental fractures, or the investigation of skeletal remains.

## Conclusions

In summary, this study investigates the method of creating 3D-printed human bone scapula and hip bone models. We successfully created 3D digital models with a 3D Slicer with maximum possible accuracy, and a few incorrect anatomical features in digital models were corrected using Meshmixer software. It was possible to produce anatomically accurate models that bore the greatest possible resemblance to the original bones and the textbook descriptions after 3D printing and post-processing. These models could be alternatives to real dry bones in medical colleges where sufficient bones are unavailable. This method could utilised by clinicians to create patient-specific models for medical education and research.
